# Responsiveness differences in outcome instruments after revision hip arthroplasty: What are the implications?

**DOI:** 10.1186/1471-2474-12-107

**Published:** 2011-05-23

**Authors:** Jasvinder A Singh

**Affiliations:** 1 Medicine Service, Birmingham VA Medical Center and Department of Medicine, University of Alabama, Birmingham, AL, USA; 2 Center for Surgical Medical Acute care Research and Transitions (C-SMART), Birmingham VA Medical Center, Birmingham, AL, USA; 3 Division of Epidemiology, School of Public Health, University of Alabama, Birmingham, AL, USA; 4 Department of Orthopedic Surgery, Mayo Clinic School of Medicine, Rochester, MN., USA

**Keywords:** Responsiveness, hip arthroplasty, harris hip score, short-form 36, SF-36, HHS

## Abstract

Responsiveness to change is an important psychometric property of an outcome instrument. Assessment of health-related quality of life (HRQoL) is critical to outcome assessment after total joint replacement, a surgery aimed at improving pain, function and HRQoL of the patients undergoing these procedures. In a recent study, Shi et al. examined the responsiveness to change of various subscales of two instruments, physician-administered Harris Hip Score and patient self-administered Short Form-36 (SF-36), 6 months after revision total hip arthroplasty. The responsiveness statistics for both scales were reasonable, higher for Harris Hip Score than SF-36. This is the first study to examine responsiveness of these instruments in revision THA patients in a systematic fashion.

## Commentary

Outcome measurement is the key to assessment of efficacy/effectiveness of joint arthroplasty, an elective procedure aimed at relieving pain and improving function, health-related quality of life (HRQoL) and mobility. Instruments used to assess HRQoL outcomes (and function) following hip arthroplasty can be physician-administered disease-specific measures such as Harris Hip Score (HHS), patient-administered generic assessments such as Short Form 36 (SF-36), patient-administered joint-specific instrument such as Oxford hip score or patient-administered limb-specific instrument such as Western Ontario and McMaster Osteoarthritis Index (WOMAC). Use of patient-reported HRQoL/function outcome measures is considered the gold standard for the assessment of patient-reported outcomes (PROs). A major National Institute of Health Initiative, the Patient-Reported Outcome Measurement Information System (PROMIS), is focused on this aspect of outcome assessment [1]. While a variety of outcome instruments are used and reported in studies of hip arthroplasty, HHS is the most commonly used instrument in studies of hip arthroplasty [2].

In a recent issue of the journal, Shi et al. assessed the responsiveness and minimal important differences (MID) [3]. Specifically, they compared two instruments, physician-administered HHS and patient-reported SF-36. Sixty-seven patients completed surveys pre-operative and 6-month post-revision total hip arthroplasty (THA). Two measures of responsiveness, i.e., the effect size (mean change from baseline to 6-months divided by the standard deviation of baseline scores) and standardized response means (ratio of mean change and standard deviation of change scores) were significantly more for HHS pain and function subscales than for the respective comparable SF-36 subscales, bodily pain and physical functioning. MID, estimated as 0.5 times standard deviation, was 2.3 for HHS physical function (range 0-44) and 3.4 for HHS pain scales (range 0-46) compared to 3.2 for SF-36 physical function (range 0-100) and 14.9 for SF-36 bodily pain (range 0-100) subscales. Several findings of this important study deserve further discussion and need to be viewed in context of published literature in this area. These findings must be interpreted while considering the study limitations including a small study sample size, potential generalizability issues to US and European populations and lack of validation data regarding the Chinese version of HHS.

This study provides comparison of responsiveness of two common HRQoL assessment tools used in the patients with revision THA, the HHS and SF-36. Use of Generalized estimating equations (GEE) technique is a particular strength of this analysis, since it allows for allows for correlation without the need for defining a model for dependency of variables. Several previous studies have examined these instruments in patients with primary THA and found that disease-specific instruments were more responsive than generic instruments [4]. The findings from this study extend these findings to the revision THA cohorts. Although, not directly comparable due to differences in study populations and their characteristics, the responsiveness statistics estimated in previous studies in primary THA [4-7] are in the same range as in this study [3]. Significant improvements in HRQoL similar to those observed in patients with primary THA have been reported in patients with revision THA. It is reassuring that same patient-reported outcome instruments are responsive in both primary and revision THA cohorts. This is a major advantage implying that similar instruments can be employed in these cohorts decreasing the variability introduced by the use of multiple instruments. As a rough guide, an effect size of 0.20-0.49 represents a small change, 0.50-0.79 a medium change, and ≥ 0.80, a large change [8]. Importantly, the effect sizes for HHS pain, HHS physical function scales and SF-36 physical functioning subscales exceeded 0.8 (large effect size) and were higher for HHS compared to SF-36. The effect size was 0.41 for SF-36 bodily pain (small effect size). A systematic review found effect sizes ranging 2.35-3.91 for physician-administered measures were higher than the effect sizes for patient-administered measures in patients who underwent total knee arthroplasty [9], that ranged 1.27-1.62. The exact reasons for greater effect sizes with physician-administered instruments are unclear. Potential explanations include patient's desire to report a better health condition when queried by the physician and physician's assessment bias. In a recent study, we found that patients report less pain in physicians' office when queried by the health care provider compared to that pain reported by them by a mailed survey completed at home [10]. Regardless of the reasons, the differences in physician versus patient-administered surveys are obvious. The current gold standard in PROs is patient-reported assessments, and therefore one can expect that use of patient-reported assessments will increase over time. Patient self-administered surveys are also more practical than a physician-administered survey, both in clinical practice and clinical research settings.

These findings should prompt more studies to examine the comparative responsiveness of other measures in revision THA patients, which might allow the discovery of the most sensitive HRQoL instruments for use in clinical trials for treatment comparisons. The responsiveness of HRQoL instruments would be expected to be higher with a surgical intervention such as revision THA surgery compared to a medical intervention for pain relief in the same cohort of patients.

The responsiveness of role limitations subscales were greater as compared to pain and physical function subscales based on effect size and standardized response mean. This is not unexpected considering that both limitation scales are single questions as opposed to multiple questions in physical functioning subscale and the pre-operative score on one role limitation subscale (role physical) was lower compared to physical function and pain subscale scores. A lower preoperative value allows for a greater chance of improvement and less of a ceiling effect. This observation is in agreement with significant and important changes noted in these scales in previous studies [7,11,12].

The study provided minimally important difference (MID) estimates based on statistical method using 0.5 times standard deviation of the mean difference. The estimation of MID is in contrast to the methods used to define minimal clinically important difference (MCID) using patient-based anchor such as patient global assessment of change. Although there is an ongoing debate whether estimates derived from a statistical approach such as 0.5 SD are similar to those derived using a patient-reported anchor-based approach, in several instances they may be very similar. A more important point to keep in mind is that MID or MCID should not be thought as an absolute number, but at best an estimate derived from a given sample. It can differ based on study setting, severity of the disease and the type of intervention being assessed. A detailed discussion of issues related to MCID assessment and other aspects of HRQoL assessments in patients with arthroplasty with a focus on problems and solutions has been recently published [13]. An approach to achieving consensus in outcome assessments in patients with arthroplasty is needed and was discussed by Riddle and colleagues in a recent publication [14].

## Conclusions: What is the take home message?

This study provides evidence that both HHS and SF-36 are responsive to change in patients undergoing revision THA and can be used in HRQoL assessment for longitudinal studies in patients with revision THA. This study also provides estimates of MID, which can be used to power future studies comparing different surgical approaches or implant types in patients undergoing revision THA, using either of these two measures as primary or secondary outcome measures. The finding that the disease-specific HHS was more sensitive to change than generic SF-36 is not surprising and should not be interpreted as a rationale to not include SF-36 in the assessment of patients with revision THA. In fact, a generic instrument such as SF-36 captures HRQoL domains differently than the disease-specific HHS, and can compliment the information obtained by the use of HHS. In addition, availability of population norms for SF-36 and availability of scores for other health conditions and chronic diseases allows comparisons of HRQoL gains across disease conditions, which plays an important role in health care policy. In conclusion, this study advances our knowledge in HRQoL assessment in patients with hip arthroplasty, and provides clinicians with tools for assessment of outcomes in clinical practice and researchers and trialists with additional data to design more robust studies in the future.

## Competing interests

There are no financial conflicts related to this work. J.A.S. has received speaker honoraria from Abbott; research and travel grants from Allergan, Takeda, Savient, Wyeth and Amgen; and consultant fees from Savient, Takeda, URL pharmaceuticals and Novartis.

## Authors' contributions

JAS: Concept of the editorial, literature review, preparation of editorial and revision

## Pre-publication history

The pre-publication history for this paper can be accessed here:

http://www.biomedcentral.com/1471-2474/12/107/prepub
